# Selected Plant-Related Papers from the First Joint Meeting on Soil and Plant System Sciences (SPSS 2019)—“Natural and Human-Induced Impacts on the Critical Zone and Food Production”

**DOI:** 10.3390/plants9091132

**Published:** 2020-09-01

**Authors:** Claudio Zaccone, Michela Schiavon, Silvia Celletti, Teodoro Miano

**Affiliations:** 1 Department of Biotechnology, University of Verona, 37134 Verona, Italy; 2 Department of Agronomy, Food, Natural resources, Animals and Environment, University of Padova, 35122 Padova, Italy; michela.schiavon@unipd.it; 3 Faculty of Science and Technology, Free University of Bozen-Bolzano, 39100 Bolzano, Italy; silvia.celletti@unibz.it; 4 Department of Soil, Plant and Food Sciences, University of Bari “Aldo Moro”, 70121 Bari, Italy; teodoro.miano@uniba.it

**Keywords:** amendment, biofortification, biostimulants, food production, nutrition, PGPR, remediation, rhizosphere, salinity, stress, sustainability

## Abstract

The First Joint Meeting on Soil and Plant System Sciences (SPSS 2019), titled “Natural and Human-Induced Impacts on the Critical Zone and Food Production”, aimed at integrating different scientific backgrounds and topics flowing into the Critical Zone, where chemical, biological, physical, and geological processes work together to support life on the Earth’s surface. The SPSS 2019 meeting gathered the thoughts and findings of scientists, professionals and individuals from different countries working in different research fields. This Special Issue comprises a selection of original works on the plant-related topics presented during this international meeting.

## 1. Introduction

The First Joint Meeting on Soil and Plant System Sciences (SPSS 2019), organized by the Italian Society of Agricultural Chemistry (SICA), the Italian Society of Pedology (SIPe) and the Italian Society of Soil Science (SISS), in collaboration with the Mediterranean Agronomic Institute of Bari (CIHEAM) and the Italian Chapter of the International Humic Substances Society (IHSS), was held on 23–26 September 2019 at the CIHEAM Campus in Valenzano (Bari, Italy).

The title of this meeting, “Natural and Human-Induced Impacts on the Critical Zone and Food Production”, mirrored the ambitious goal of integrating different scientific backgrounds and topics flowing into the Critical Zone, where chemical, biological, physical, and geological processes work together to support life on the Earth’s surface [1]. In fact, traditionally, the different aspects of the Critical Zone have usually been studied by scientists in distinct and often isolated disciplines, e.g., vegetation by biologists and plant physiologists, soils by soil scientists, microorganisms by microbiologists, rocks by geologists. On the other hand, in a more modern, holistic approach, vegetation, soils, microorganisms and landforms are all integral parts of the Critical Zone, an open system that exchanges, at various scales, matter and energy with the atmosphere, lithosphere, biosphere and hydrosphere, and regulates life-sustaining resources [2,3,4].

To meet humankind’s growing needs for food, fiber, and bioenergy, crop and soil productivity will be vastly expanded in the next few decades, with substantial effects on the Critical Zone [5]. Therefore, during the Anthropocene, the Critical Zone is increasingly being affected by natural processes and human activities (e.g., land use intensification, climate change, desertification, pollution) [2,6,7,8,9,10], with consequences for food production, safety and security [9].

The scientific program of the SPSS 2019 meeting consisted of five sessions, i.e., (i) natural and agricultural soil systems, (ii) soil pollution and food safety, (iii) organic amendments and soil quality, (iv) plant responses to natural and human-induced drivers, and (v) frontiers in plant and soil sciences.

The SPSS 2019 meeting gathered the thoughts and findings of scientists from several scientific societies and institutions involved in research approaches to the Critical Zone. More than 160 scientists, professionals and individuals from different countries (including Italy, Spain, Greece, Russia, United Kingdom, Germany, Nigeria and Trinidad and Tobago) attended the meeting; 35 oral presentations and 90 posters contributed to creating a permanent forum of stimulating scientific debates, especially for young scientists and students.

This Special Issue comprises a selection of original papers on the plant-related topics presented during this international meeting.

## 2. Highlights of the Special Issue

Puglisi et al. [11] studied whether the use of different concentrations of microalgae extracts, such as those of *Clorella vulgaris* and *Scenedesmus quadricauda*, could improve the germination process of sugar beet (*Beta vulgaris* subsp. *vulgaris*). While the quality of the seeds is essential for proper plant growth and production [12], the productivity of sugar beet is often limited by the heterogeneity of germination in the field [13,14]. The results show that microalgae extracts can be used as a priming agent, as they increase the efficiency and regularity of the germination process of *B. vulgaris* seeds. The best results, in terms of germination indices as well as the morphological traits of the roots, were achieved using *C. vulgaris* extract at concentrations of 1 and 2 mg C_org_ L^−^^1^. This suggests that *C. vulgaris* extract may represent a promising practice to increase the physiological potential of sugar beet seeds.

Mbarki et al. [15] discussed the salinity tolerance of the genotypes of *Medicago ciliaris*, an annual legume whose adaptation to agroclimatic conditions has not yet been well described, compared to *Medicago intertexta* and *Medicago scutellata*. Salt accumulation in soils modifies the plant’s physiology and metabolism, and negatively affects the germination and growth of seedlings, with a consequent decrease in yields and the quality of crop production [16,17,18]. Therefore, in this study, salt tolerance was investigated through physiological and biochemical characterization both at germination and seedling early growth stages. The results show that three *M. ciliaris* populations (*Pop1*, *355* and *667*), among the seven studied, were more salt tolerant and could be the most promising for germination and growth under moderate salt stress. On the other hand, two *M. ciliaris* populations (*306* and *773*) and *M. scutellata* ranged from being moderately tolerant to sensitive to salt stress depending on the salt rates. These findings can be exploited as an efficient strategy to reveal genetic variations in response to salt stress. Moreover, this approach allowed the selection of desirable traits, promoting more effective applications of breeding methods to obtain stress-tolerant *M. ciliaris* populations.

Benjamin et al. [19] investigated the salinity-induced protein variations (proteomic approach) in two halophyte species, *Salicornia brachiata* Roxb and *Suaeda maritima* (L.) Dumort, which are known to be salt accumulators and have the capacity to adapt to high salinity levels (200 mM NaCl) [20,21,22]. Plant resistance to salinity stress is a major challenge in agriculture; thus, understanding the molecular and cellular mechanisms involved in plant tolerance can help counteract crop losses due to high-salinity soil conditions [23,24,25]. In order to highlight the key processes involved in the tolerance of these plants to NaCl, the authors conducted an analysis of the plant proteome, which is a powerful tool to shed light on the physiological response to environmental stress, since proteins are the main regulators of cellular responses [26]. In this study, two distinct protein profiles were observed between the two halophytes under NaCl salinity, suggesting different mechanisms of NaCl tolerance in *S. brachiata* and *S. maritima*, with *S. brachiata* having a higher tolerance to salinity than *S. maritima*.

Santoro et al. [27] investigated the role of an emerging class of phytohormones, namely strigolactones (SL), on root anatomy and system architecture traits of wild-type and strigolactone-depleted tomato plants grown under different phosphorus (P) foraging conditions. Strigolactones are involved in root–rhizosphere interactions by promoting mycorrhiza symbiosis establishment under P limitation, and mediate the signaling networks in plants that crosstalk with those of other phytohormones during P perception and signal transduction [28,29]. The results from this study confirm the role of SL in mediating plant acclimation responses to varying nutritional P levels in tomato plants, and show that this class of molecules modulates a number of root traits, especially primary and total root elongation, lateral root number, root volume and diameter, which support greater soil exploration by the roots. Phenotypic differences between wild-type and mutant plants were also evident in terms of root phenotype and tip anatomy. In particular, a condition of hypersensitivity to P deprivation stress was observed in the tips of strigolactone-depleted roots, as revealed by significant alterations in cell differentiation processes and tissue organization. Thus, the manipulation of SL biosynthesis could modify the root system architecture in order to obtain plants with root traits that are more suitable for adaptation to low-P conditions.

Research by Puccinelli et al. [30] aimed to biofortify basil plants with selenium (Se) and analyze the effects of Se enrichment on the growth, quality and nutritional value of basil leaves at harvest and after a few days of storage. Selenium biofortification of crops is getting increasing recognition and has been attained in several countries worldwide to combat nutritional deficiency in vulnerable populations [31,32], given that this element is essentially required by humans and animals for a healthy metabolism [33]. Moreover, Se is a beneficial element to many plants, as it can trigger positive effects on plant metabolism under hostile conditions and induce antioxidant responses in cells when applied in trace amounts [34,35]. In this study, the application of increasing Se doses (4, 8 and 12 mg L^−1^) was effective at enriching basil leaves in Se, even if this only occurred in the first cut. Interestingly, Se enhanced the antioxidant capacity and the content of total phenols and rosmarinic acid in plants at harvest. Furthermore, the reduction in the content of ethylene observed in plants treated with 4 mg Se L^−1^ after five days of storage was indicative of the capacity of Se to delay plant senescence and increase the shelf-life of basil. Therefore, Se was beneficial to basil, preventing oxidative and senescence-related processes during basil leaf storage.

De Bernardi et al. [36] directed their research toward testing the phytoremediation potential of four crop species for nickel (Ni) in order to sustain the application of green clean-up techniques suitable for the remediation of Ni-contaminated sites. The legal threshold for water-soluble Ni in soil, set by Italian Legislative Decree 186/2006, is 10 µg L^−1^ [37]. Therefore, successful phytoremediation technology is expected to reduce Ni concentrations below this limit. Nickel is an essential trace nutrient for plants, but becomes potentially toxic at high concentrations, owing to its capacity to elicit oxidative stress responses and accumulate in the ecosystem [38]. Plants used in Ni phytoremediation should be moderately or highly tolerant of Ni. The authors assayed two autumn–winter species, i.e., spinach and canola, in a greenhouse study, either with or without the use of bentonite, and two spring–summer species, i.e., sorghum and sunflower, in an outdoor experiment, and found that the plants were all moderately tolerant of Ni. Plants were grown on carbonation lime, a waste from the sugar industry made up of 90% calcium carbonate. Such a substrate contained 15 and 331 µg L^−1^ for soluble and bioavailable Ni fractions, respectively. The results from this study indicate that plants in the greenhouse reduced Ni below the Italian legal limit and the addition of bentonite decreased the amount of Ni in lime, mainly in the bioavailable fraction. Spinach and sunflower displayed a higher phytoextraction potential for Ni, while the outdoor experiment pointed to sorghum as a good candidate for Ni phytostabilization due to its capacity to accumulate Ni mostly in the roots and to retain a significant amount of bioavailable Ni in the rhizosphere.

Guerrieri et al. [39] highlighted the potential of bacteria isolated from the rhizosphere when processing tomato plants after long-term reduced tillage and cover crop soil management in terms of both their growth-promoting activity and biocontrol of the fungal phytopathogen *Sclerotinia sclerotiorum*. It must be noted that plant growth-promoting rhizobacteria (PGPR) are gaining increasing relevance among biostimulants to reduce the application of agrochemicals in the framework of sustainable agriculture and tackle crop yield losses due to ongoing climate change [40,41]. The bacteria isolated in this study were first evaluated for their capacity to fix nitrogen. Selected strains were clustered further based on DNA sequence profile commonalities and subjected to various screening tests to assay their capacity to promote plant nutrition and their effects against phytopathogenic fungi. All strains exhibited at least one plant growth-promoting property (e.g., indoleacetic acid production, siderophore production, phosphate solubilization) and an antifungal capacity. In particular, two strains, UC4094 and UC4098, were able to exert all the promoting properties examined and came out on top in the rankings of each strain. However, their properties were expressed to a lesser extent compared to other strains that expressed only one property but at a higher level. These findings suggest that developing a bacterial consortium that includes isolates with growth-promoting activity might be a convenient choice to meet the challenges and requirements of increasingly sustainable agriculture.

Guzzetti et al. [42] evaluated the impact of conventional tillage and no-tillage management combined with the usage of a set of cover crops, coupled with normal and deficient water regimes, on the growth performance and nutritional profile of cowpea (*Vigna unguiculata* L. Walp), commonly known as black-eyed bean, under field conditions. Conservation Agriculture (CA), based on the adoption of these sustainable agricultural practices, allows us to obtain satisfactory production yields and, in reducing carbon dioxide (CO_2_) emissions, to mitigate climate change, which puts the productivity and nutritional values of crop yields at risk due to unpredictable rainfall and water shortages [43,44]. The results obtained confirm CA’s capability to enrich surface soil horizons and reveal that cowpea is a suitable crop for cultivation not only to preserve soils, reduce agriculture’s impact and mitigate climate change, but also to provide plant-based nutrients, such as proteins and amino acids, which are important for the human diet.

In line with the need to find new management strategies to increase crop yield while preserving soil properties and reduce CO_2_ emissions into the atmosphere, Mulè et al. [45] fertilized durum wheat plants with a soil organic amendment produced through an advanced process for the non-fermentative, fast decomposition of animal byproducts (ABP). The effects induced by this bio-fertilizer on plant productivity-associated parameters were compared with those determined by the application of non-organic fertilizers, compost and no fertilization. The study was conducted for one year only, but it offered promising results, as ABP fertilization allowed the researchers to obtain taller plants with greater vigor, heavier spikes, and better grain quality when compared to both non-fertilized and nitrogen-fertilized plants. Nevertheless, grain yield did not show any variation between treatments. Although this is a preliminary study, it suggests that the application of ABP can be an effective approach for ameliorating soil properties and accomplishing high crop quality and yield under Mediterranean conditions, although further studies are needed to verify the effectiveness of ABP over a long-term period.

In view of a futuristic scenario that foresees the need for the self-sustenance of colonies on Mars due to constraints related to the delivery of food resources from Earth, Duri et al. [46] attempted to grow two lettuce cultivars (green and red Salanova^®^) on Mojave Mars regolith simulant (MMS-1) mixed with different compost percentages (0:100, 30:70, 70:30 and 100:0; *v:v*) inside a phytotron open gas exchange growth chamber. The addition of organic residues to MMS-1 ameliorated the quality of the substrate, thus evoking a better crop quality and higher yield. Indeed, the authors found that lettuce quality and performance were improved when plants thrived on a 30:70 MMS-1/compost mixture than on MMS-1 alone. The main quality traits that were enhanced, especially in the red cultivar, included photosynthetic activity and the content of bioactive compounds (ascorbic acid and total polyphenols). The 70:30 MMS-1/compost mixture provided less benefits to plants than the 30:70 MMS-1/compost mixture, but likely accounted for a more realistic substrate to exploit on Mars, owing to the restricted use of compost as a resource in space farming. On the whole, this study highlights the importance of enriching Mars regolith with organic residues to increase its fertility, but also suggested that future experiments should additionally address either crop cultivation without additive fertigation and solely considering in situ substrate fertility, or alternative organic sources such as human waste.
